# Acute stromal keratitis in clinics – are we missing microsporidia?

**DOI:** 10.3205/oc000128

**Published:** 2020-02-14

**Authors:** Javed Hussain Farooqui, Manisha Acharya, Arpan Gandhi, Umang Mathur

**Affiliations:** 1 Cornea, Cataract and Refractive Surgery, Dr. Shroff’s Charity Eye Hospital, New Delhi, India; 2 Laboratory Services, Dr. Shroff’s Charity Eye Hospital, New Delhi, India

**Keywords:** microsporidial stromal keratitis, microsporidia

## Abstract

**Purpose:** To report 3 cases of microsporidial stromal keratitis presenting as a diagnostic dilemma to a tertiary eye care center in north India.

**Methods:** Three eyes of 3 patients underwent therapeutic keratoplasty for microsporidial stromal keratitis. A decision for early surgery was taken as the patients were not responding to conventional medical management and were worsening clinically. The diagnosis of microsporidia was made by corneal scraping and confirmed on histopathological evaluation of the corneal button.

**Results:** Out of the 3 patients, one maintained a clear graft, one had a recurrence and one had graft rejection, 6 months postoperatively. The patients were not started on steroids in the postoperative period and were given topical antibiotics and polyhexamethylene biguanide (PHMD). Oral Albendazole 400 mg was also given twice a day for a month.

**Conclusion:** Many questions remained unanswered about the management protocol of stromal keratitis caused by microsporidia. The role of topical steroids, antifungal agents, oral Albendazole needs to be discussed. Clinicians should be aware of recurrences which may mimic as rejections. There needs to be more awareness regarding microsporidia as a cause of acute stromal keratitis, so that its not overlooked or underdiagnosed.

## Introduction

Since the time it was first described by Ashton [1] in 1973 as a cause of keratitis, microsporidiosis has come a long way as being recognized as a potential sight-threatening organism. In literature, the pattern of involvement has been described as either keratoconjunctivitis in immunocompromised individuals or stromal keratitis in immunocompetent individuals [2]. Even though the treatment of keratoconjunctivitis is straightforward, the stromal keratitis variant poses a diagnostic and therapeutic challenge. Often misdiagnosed as herpes simplex keratits [3], it also closely mimics bacterial or fungal keratitis, with no specific ulcer pattern having been described till date [4]. We report a series of 3 eyes of 3 patients who presented to us with acute stromal keratitis and were eventually diagnosed with microsporidial stromal keratitis and treated accordingly. To the best of our knowledge, no major case series of microsporidial stromal keratitis has been described from north India. 

## Case descriptions

### Case 1

**Figure 1:**
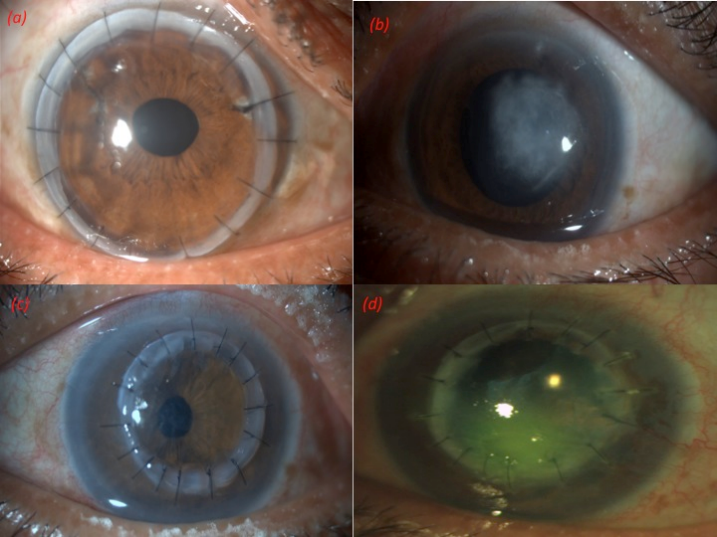
(a) Right eye showing a compact graft, with intact sutures and well-formed anterior chamber. (b) Left eye showing epithelial defect with whitish grey midstromal infiltrate in the mid periphery measuring 5 mm x 5 mm with ill-defined margins. (c) One week post therapeutic penetrating keratoplasty. (d) Six months postoperatively, midstromal infiltrate measuring 4 mm x 4 mm with an overlying epithelial defect.

A 73-year-old male patient presented with pain, watering, photophobia, and blurring of vision in the right eye since one year and in the left eye since 4 months. He gave history of a foreign body sensation, which commenced by itself with no history of trauma or recurrence in the left eye. He consulted a tertiary eye center for his right eye and records revealed a presence of keratitis with posterior segment involvement for which penetrating keratoplasty (PKP) with pars plana vitrectomy in the right eye was done and vitreous biopsy confirmed the presence of microsporidial spores. His unaided visual acuity at presentation was 20/200 in the right eye and 20/120 in the left eye. Slit lamp examination of the right eye showed a 10.5 mm clear, compact graft with intact sutures and a well-formed anterior chamber (Figure 1a (Fig. 1)). Posterior chamber intraocular lens was noted with glaucomatous optic atrophy. The left eye examination showed an epithelial defect with a whitish grey midstromal infiltrate in the mid periphery measuring 5 mm x 5 mm with ill-defined margins (Figure 1b (Fig. 1)). Corneal sensations were intact. The anterior chamber had 1+ flare and cells. Intraocular pressure was considered normal on digital tonometry. Corneal scraping and microbiological work-up was done which revealed microsporidial spores. The patient was started on polyhexamethylene biguanide (PHMB) 0.02% 2-hourly along with lubricating and cycloplegic eye drops. When the patient didn’t improve over a 1-week period, a decision of early therapeutic PKP was taken and 7.5 mm donor graft was used (Figure 1c (Fig. 1)). The surgery was uneventful and the patient was continued on PHMB and 0.5% moxifloxacin eye drops 2-hourly after the surgery. In the subsequent months, unaided visual acuity improved to 20/200 in the left eye. Unfortunately, 6 months postoperatively, the patient presented with hand movement vision and corneal scraping revealed a recurrence of microbiologically proven microsporidial keratitis with retinal detachment (Figure 1d (Fig. 1)).

### Case 2

**Figure 2:**
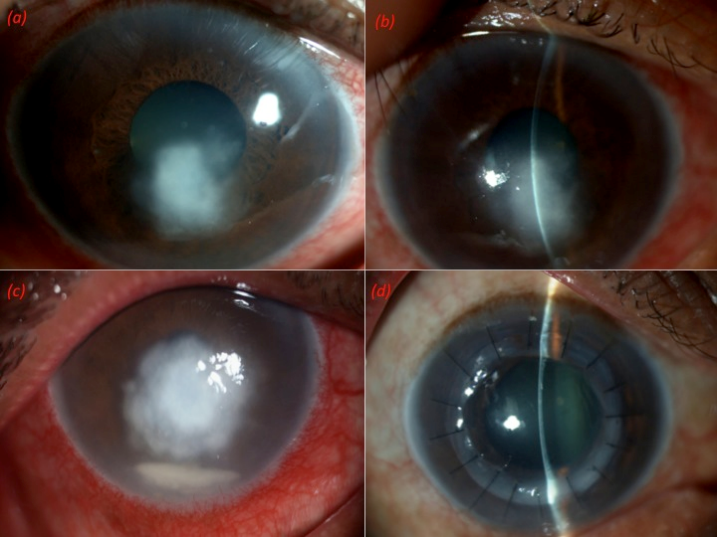
(a) Right eye showing mid stromal greyish infiltrate measuring 4 mm x 3 mm with intact overlying epithelium. (b) Third day picture with increase in the size of the infiltrate. (c) One week picture showing appearance of hypopyon along with the preexisting infiltrate. (d) Six months postoperative picture showing clear graft.

A 55-year-old male patient presented in 2018 with pain, watering, photophobia, and blurring of vision in the right eye since 1 week. He had had a similar episode 3 weeks back and had been treated with anti-virals elsewhere. His symptoms had improved and subsequently he had discontinued treatment. His unaided visual acuity was 20/200 in the right eye and 20/40 in the left eye. Slit lamp examination of the right eye showed a midstromal greyish infiltrate measuring 4 mm x 3 mm with intact overlying epithelium (Figure 2a (Fig. 2)). Corneal scraping was inconclusive and the patient was started on antivirals based on clinical suspicion. The patient did not improve on his day 3 follow-up (Figure 2b (Fig. 2)) and further worsened on day 7 with increase in the infiltrate size and appearance of a hypopyon (Figure 2c (Fig. 2)). Repeat scrapings were done on day 3 and day 7. The second scraping also did not aid in diagnosis and there was no growth on culture as well till then. A ‘drug holiday’ was given with the clinical suspicion of an atypical organism. This was confirmed on subsequent scraping, wherein microsporidial spores were identified. The patient underwent therapeutic PKP with a 8 mm graft. The postoperative period was uneventful with the patient maintaining a clear graft and a visual acuity of 20/120 over a period of 6 months postoperative (Figure 2d (Fig. 2)). 

### Case 3

**Figure 3:**
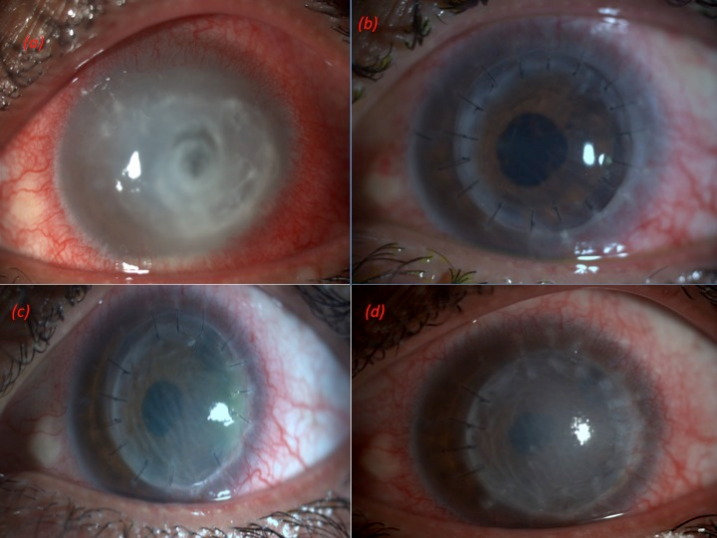
(a) Left eye showing epithelial defect 7 mm x 7 mm, multifocal sub epithelial infiltrates and central 4 mm x 4 mm with a hypopyon of 0.5 mm. (b) Post therapeutic PKP, 8 mm eccentric graft at 1-week follow-up. (c) Fourth month postoperative picture showing stromal edema and signs of graft rejection. (d) Six months postoperative picture showing failed graft.

A 22-year-old male patient presented with pain, watering, photophobia, and blurring of vision in the left eye for the last 3 weeks. His visual acuity in the right eye was 20/20 and in the left eye was counting fingers at 2 meters. Slit lamp examination of the left eye showed an epithelial defect of 7 mm x 7 mm, multifocal subepithelial infiltrates and central 4 mm x 4 mm thinning of 70%. The anterior chamber was formed with a hypopyon of 0.5 mm (Figure 3a (Fig. 3)). A working diagnosis of viral keratitis was made and antivirals were started. When the patient didn’t respond to treatment after 1 week, corneal scraping was done which revealed microsporidial spores. The patient was taken up for early therapeutic PKP, and an 8 mm eccentric graft was taken (Figure 3b (Fig. 3)). The patient did well for the first 3 months, but returned in the fourth month with fall in vision, graft edema and signs of rejection (Figure 3c (Fig. 3)). There was no infiltrate and high dose intravenous steroids were given for 3 days and topical steroids stepped up to hourly interval. Graft edema did not subside at 6 months (Figure 3d (Fig. 3)), and the patient was then given the option of repeat keratoplasty. The patient has not yet consented to the procedure.

### Postoperative regimen

All 3 patients were given 1% prednisolone acetate 6 times a day along with a cycloplegic and 0.5% moxifloxacin 4 times a day in the initial period. The first patient was prescribed 0.2% PHMB for the first month at a 2-hourly interval. The other two patients did not receive PHMB because of availability issues. Oral Albendazole 400 mg twice a day was given for the first 3 weeks after surgery. 

### Corneal scraping and laboratory work-up

**Figure 4:**
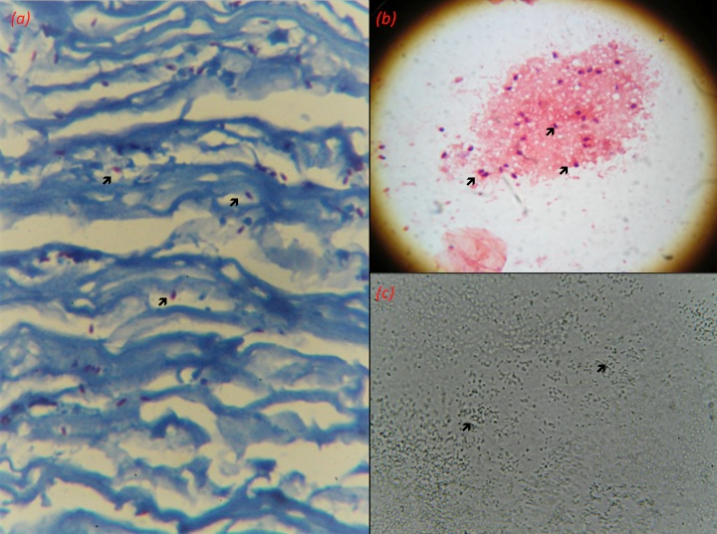
(a) Microsporidial spores (black arrow) seen as well-defined oval reddish bodies on Ziehl Neelsen stain of the corneal button derived from the first patient. (b) Gram’s stain of the second patient showing microsporidial spores (black arrow). (c) Potassium hydroxide (KOH) 10% depicting well defined microsporidial spores (black arrow), obtained from corneal scraping from the third patient.

As protocol, all the patients underwent corneal scrapings according to standard techniques mentioned in the text [5]. Slides were sent for Gram’s, Giemsa and potassium hydroxide 10%. The material was also inoculated on fresh blood agar, chocolate agar, Saburaud’s dextrose agar (SDA), non-nutrient agar with an overlay of *Escherichia coli* and brain heart infusion broth. The corneal buttons derived from surgery were also sent for microbiological and histopathological evaluation. Figure 4 (Fig. 4) shows pictures of various laboratory slides derived from the three patients.

## Discussion

India is home to a quarter of the world’s blind population [6]. Various studies have estimated corneal blindness to account for between 3 to 14% of total blindness in India, and almost 59% of this is because of keratits and its sequel [7], [8], [9]. It is to our surprise that out of such a high number of keratitis cases being reported from India, no major series of microsporidia has been reported from north India. All the major series are from south India from limited centers only [10], [11]. This prompts us to think that this condition is being misdiagnosed or missed altogether. Due to the paucity of reports, we still don’t have treatment protocols set up for treating patients with microsporidial stromal keratitis. In our patients, we were unsure about the use of topical steroids in the postoperative period. Since microsporidia has recently been classified as a fungus [3], the use of topical steroids can be debated. We did not start any of our patients on topical antifungals and wonder if there is a role of these drugs in long-term safety and graft survival. Also, PHMB has been described in previous reports [11], but its limited availability has hampered its use in clinical practice. Even though it’s available in big cities and towns, efforts need to be made to make them easily available in smaller cities as well. Along with this, clear guidelines should be set for the use of oral Albendazole. Its role in systemic microsporidiosis has been established, but most of the studies mention no benefit to ocular pathology with the use of oral Albendazole [10]. We feel unnecessary overmedication of patients is not warranted and work should be done to assess the efficacy of Albendazone for ocular microsporidiosis. 

Our last patient went into rejection at 3 months. We cannot, with certainty, say if that patient had rejection or recurrence of the disease. Microsporidiosis is known to have recurrence, and may mimic as rejection [3]. In such cases, as a repeat procedure, a full thickness graft would be better than endothelial keratoplasty to avoid recurrence. In our first and third case, a 1 mm clear margin was taken all around the infiltrate during host trephination, but this seems inadequate for microsporidia and both patients had recurrence. For the second patient, a clear margin of more than 1 mm was kept and perhaps this led to his graft survival. Although there is no way to confirm this hypothesis, these are points that should be kept in mind while operating on these patients. 

Many questions still remain unanswered about microsporidial stromal keratitis. We hope that this report provokes thought and discussion and helps us understand the disease better. We also look forward to ophthalmologists formulating universal treatment protocols for the management of this disease.

## Notes

### Competing interests

The authors declare that they have no competing interests.

### Acknowledgments

We appreciate the help of Dr. Vaibhav Pednekar and Dr. Mitali Kekan in patient work-up and data gathering.
